# Supplementary material for: Feasibility and acceptability of GeneXpert MTB/XDR implementation among healthcare workers in three low-middle income African countries
Source: PLoS One. 2025 Jun 24;20(6):e0326342. doi: 10.1371/journal.pone.0326342 (PMC12186877; doi:10.1371/journal.pone.0326342)
Supplement: S1 File — (DOCX) [file pone.0326342.s001.docx]

**Supporting Information**

File S1

Contents

[1. Interview topic guide – Lab Technicians 2](#_Toc194503763)

[2. Interview topic guide – Nurses/Clinicians 4](#_Toc194503764)

[3. Interview topic guide – Patients 6](#_Toc194503765)

[4. Interview topic guide – Policymakers 8](#_Toc194503766)

# 1. Interview topic guide – Lab Technicians

For this recording, do you agree to take part in this interview?

Do you agree with this interview being recorded?

*Make sure both questions are answered before continuing!*

Before we start the interview, I would like you to provide me with some demographic information. Could you please tell me your…?

**Demographics**

1. Age:
2. Sex:
3. Male
4. Female
5. Other (please fill in):
6. Don’t want to tell
7. Which country:
   1. South Africa
   2. Nigeria
   3. Ethiopia
8. Which study site/health care facility *only for administrative reasons (will be deleted afterwards)*:
9. **Hospital 1**, South Africa
10. **Hospital 2**, South Africa
11. **Hospital 1**, Nigeria
12. **Hospital 2**, Nigeria
13. **Hospital 1**, Ethiopia
14. **Hospital 2**, Ethiopia
15. **Hospital 3**, Ethiopia
16. **Hospital 4**, Ethiopia
17. **Hospital 5**, Ethiopia
18. **Hospital 6,** Ethiopia
19. **Hospital 7,** Ethiopia
20. **Hospital 8,** Ethiopia
21. **Other:** please name
22. Which occupation within a health care facility (MC)?
23. Nurse
24. Doctor/clinician
25. Laboratory staff member
26. Study site coordinator
27. Other: please specify

Introduction:

*Thank you for taking the time to talk with me. The focus of our discussion will be on how you use rapid triaging/Xpert MTB/XDR in daily practice. The information you provide will remain completely confidential and will be helpful to improve future rapid triaging/Xpert MTB/XDR.*

Opening:

| Getting to know the interviewee | - What is your role in the clinic?   - How long have you been working in TB?   - What other areas of care have you worked in? - Can you walk me through a typical day   - Are there other TB diagnostic areas they work with |
| --- | --- |
| Xpert MTB/XDR Knowledge  (intervention coherence & opportunity cost) | - Can you please explain your role with Xpert *MTB/XDR*   - Walk me through the steps   - How are patients referred to you(referral from another clinic, clinician referral, where are they referred from)   - How do you document these different stages - How was Xpert XDR was implemented in your lab   - When/who/how were you informed   - What was it like before Xpert XDR was implemented - Walk me through the training process for Xpert XDR   - How long was the training, how effective did you find the training   - Do you feel confident in this new pathway |
| Xpert MTB/XDR Experience  (affective attitude, self-efficacy, burden) | - What are some of the facilitators or benefits in implementing Xpert XDR into your workflow   - What’s been a positive, what’s worked   - Ex. Faster turnaround time, one test for multiple resistance types   - Benefits compared to the previous method of drug resistance pattern testing - What are some of the challenges you’ve faced with Xpert XDR   - Ex: Inconclusive results, discordant results, power challenges, not enough sputum, interpreting results   - Challenges compared to previous method of drug resistance pattern testing   - Is there anything you would change - Does using the combination of Xpert MTB/RIF Ultra and XDR have an impact on the speed of your diagnosis   - Y/N/ how and why do you think that - Can you describe how you explain the process to patients (ETH only)   - What do they inform them about   - Who shares the results with the patient   - How does information get back to the provider |
| Final Questions  (ethics) | - Would you recommend Xpert XDR to other healthcare facilities or labs?   - Why, why not |

# 2. Interview topic guide – Nurses/Clinicians

For this recording, do you agree to take part in this interview?

Do you agree with this interview being recorded?

*Make sure both questions are answered before continuing*

Before we start the interview, I would like you to provide me with some demographic information. Could you please tell me your…?

**Demographics**

1. Age:
2. Sex
3. Male
4. Female
5. Other (please fill in):
6. Don’t want to tell
7. Which country (MC):
   1. South Africa
   2. Nigeria
   3. Ethiopia
8. Which study site/health care facility (MC) *only for administrative reasons (will be deleted afterward)*:
   1. **Hospital 1**, South Africa
   2. **Hospital 2**, South Africa
   3. **Hospital 1**, Nigeria
   4. **Hospital 2**, Nigeria
   5. **Hospital 1**, Ethiopia
   6. **Hospital 2**, Ethiopia
   7. **Hospital 3**, Ethiopia
   8. **Hospital 4**, Ethiopia
   9. **Hospital 5**, Ethiopia
   10. **Hospital 6,** Ethiopia
   11. **Hospital 7,** Ethiopia
   12. **Hospital 8,** Ethiopia
   13. **Other:** please name
9. Which occupation within a health care facility (MC)?
10. Nurse
11. Doctor/clinician
12. Laboratory staff member
13. Study site coordinator
14. Other: please specify

Introduction:

*Thank you for taking the time to talk with me. I want to learn more about your experiences with rapid triaging and/or the use of Xpert MTB/XDR. The focus of our discussion will be on how you use rapid triaging/Xpert MTB/XDR in daily practice. The information you provide will remain completely confidential and will be helpful to improve future rapid triaging/Xpert MTB/XDR.*

Opening:

| Getting to know the interviewee | - What is your role in the clinic   - How long have you been providing TB care?   - What other areas of care have you worked in prior to this one - Can you walk me through a typical day |
| --- | --- |
| Xpert MTB/XDR Knowledge  (intervention coherence & opportunity cost) | - Can you please explain your role with rapid triaging using Xpert MTB/XDR   - Walk me through the steps   - How do you document these different stages   - How do you communication with the lab   - How is it implemented in your daily workflow - How was rapid triaging/Xpert XDR implemented in your clinic   - When/who/how were you informed   - Were you trained? How effective was the training   - Do you feel confident with this new process - How is this new method of rapid triage different than what was done before?   - Which method do you prefer and why?   - How does it impact the speed of diagnosis |
| Xpert MTB/XDR Patient conversations  (intervention coherence) | - How do you explain this new triaging process to patients   - Walk me through the patient pathway - How are results communicated to the patient   - Do patients wait in the clinic, how are they notified of the results - What impact does XDR have on patients |
| Xpert MTB/XDR Experience  (affective attitude, self-efficacy, burden) | - What are some of the benefits to the new Xpert XDR workflow?   - Ex: faster time to treatment initiation - What are some of the barriers to the new Xpert XDR triaging workflow?   - Ex: dealing with discordant or inclusive results   - Is there anything you would change |
| Final Questions  (ethics) | - Would you recommend Xpert XDR or the new rapid triage process to other health care facilities?   - Why, why not |

# 3. Interview topic guide – Patients

For this recording, do you agree to take part in this interview?

Do you agree with this interview being recorded?

*Make sure both questions are answered before continuing*

Before we start the interview, I would like you to provide me with some demographic information. Could you please tell me your…?

**Demographics**

1. Age:
2. Sex
3. Male
4. Female
5. Other (please fill in):
6. Don’t want to tell
7. Which country:
   1. South Africa
   2. Nigeria
   3. Ethiopia
8. Which study site/health care facility: *only for administrative reasons (will be deleted afterwards)*:
   1. **Hospital 1**, South Africa
   2. **Hospital 2**, South Africa
   3. **Hospital 1**, Nigeria
   4. **Hospital 2**, Nigeria
   5. **Hospital 1**, Ethiopia
   6. **Hospital 2**, Ethiopia
   7. **Hospital 3**, Ethiopia
   8. **Hospital 4**, Ethiopia
   9. **Hospital 5**, Ethiopia
   10. **Hospital 6,** Ethiopia
   11. **Hospital 7,** Ethiopia
   12. **Hospital 8,** Ethiopia
   13. **Other:** please name
9. Income level: *Circle Correct Income*

|  | **South Africa** | **Ethiopia** | **Nigeria** |
| --- | --- | --- | --- |
| A | ≤2,000 R/month | no income | ≤30,000 N/month |
| B | >2,000 – ≤5,000 R/month | >0 – ≤1500 birr/month | >30,000 – ≤100,000 N/month |
| C | >5,000 – ≤8,000 R/month | >1500 – ≤ 5000 birr/month | >100,000 – ≤200,000 N/month |
| D | >8,000 R/month | >5000 birr/month | >200,000 N/month |

1. Which diagnosis (if the patient does not know, try to collect from HCW, patient file, or treatment card): *Circle Correct Diagnosis*

| DR-TB | Hr-TB | Pre-XDR-TB |
| --- | --- | --- |
| Rr-TB | MDR-TB | XDR-TB |

Introduction:

*Thank you for taking the time to talk with me. I want to learn more about your experiences with at this healthcare facility from testing to treatment. There is a new triaging process at this clinic designed to improve the time in diagnosing TB and identifying drug resistance, so that you can people with TB can be put on the right medication regimen sooner. Your participation in this conversation is completely voluntary and may be stopped at any time. The information you provide will remain completely confidential and is helpful in understanding the patient perspective of rapid triaging.*

| Getting to know the interviewee | - Tell me about what made you first decide to come to the healthcare facility/hospital   - What were your complaints   - Did you first seek care at this site or another, what was done there?   - What led you to be tested for TB, did you have a say in this |
| --- | --- |
| Referral  *(skip if the patient didn’t receive a referral to this clinic)* | - Did you get a referral to this clinic? (if no skip)   - Can you walk me through the referral process   - When were you referred? - How did it feel if you were unable to get care at your home clinic? - How long did it take to get your appointment at this clinic   - How did the in between time feel? |
| Diagnosis | - Can you walk me through your first visit to this facility?   - How was the experience for you - How was the testing/diagnosis process for you   - Sputum collection – how was that process   - Did you face any challenges (pain, inability to produce sputum) during sputum collection?   - How was it explained to you/did you understand the process   - What were you told to expect during this process - How long did it take you to receive your results   - How did it feel to wait?   - Did you need to come back multiple times   - How were you informed of the results |
| Treatment | - How long was it from the time you were told the results to the time you were put on a treatment plan?   - How did that feel   - What are some of the things he/she did that made you feel that way   - How long have you been on treatment |
| Experience | - Do you feel that your clinician had enough time for you during this entire process - Do you have recommendations on what could be improved on from process of testing to treatment |

# 4. Interview topic guide – Policymakers

For this recording, do you agree to take part in this interview?

Do you agree with this interview being recorded?

*Make sure both questions are answered before continuing*

Before we start the interview, I would like you to provide me with some demographic information. Could you please tell me your…?

**Demographics**

1. Age:
2. Sex:
3. Male
4. Female
5. Other (please fill in):
6. Don’t want to tell
7. Which country:
   1. South Africa
   2. Nigeria
   3. Ethiopia
8. Which level:
9. National
10. Regional
11. District
12. Local

Introduction:

*Thank you for taking the time to talk with me. I want to learn more about your experiences with rapid triaging and/or the use of Xpert MTB/XDR. The focus of our discussion will be on how rapid triaging/Xpert XDR was implemented in the study sites and the potential for implementation on a larger scale*. *The information you provide will remain completely confidential and will be helpful to improve future rapid triaging/Xpert MTB/XDR*. *Do you have any questions before we start?*

| Getting to know the interviewee | - Can you please tell me about yourself and what your role with TB - How long have you been working in this role? |
| --- | --- |
| Xpert MTB/XDR Knowledge | - Can you tell me what you know about Xpert MTB/XDR?   - How did you hear about it/what is your opinion on it   - What do you know about the new workflow |
| Xpert MTB/XDR  Implementation | - How were you involved in the implementation?   - Directly or indirectly   - Who else was involved - Can you walk me through the steps that you took at the programmatic/policy level to implement rapid triaging/ Xpert XDR in clinics? - What are some the facilitators in creating new policies around TB workflows and tools like Xpert XDR?   - Time, labor, etc - What are some the barriers in creating new policies around TB workflows and tools like Xpert XDR?   - Stockouts of cartridge, electricity cut outs, etc.   - How did you overcome these barriers? - What factors have influenced the performance of rapid triaging in *the South African/Nigerian/Ethiopian* health system? |
| Larger policy questions | - What is needed to successful embed rapid triaging/Xpert XDR into clinic workflows?   - What factors can impact this? Ex finances - To what extent do you think it could improve TB care in your country/region/province?   - What is needed on a daily basis?   - Practical improvements? Ask for examples |
| Final Questions  (ethics) | - Would you recommend Xpert XDR or the new rapid triage process to other health care facilities?   - Why, why not |
