# Supplementary material for: Feasibility and acceptability of GeneXpert MTB/XDR implementation among healthcare workers in three low-middle income African countries
Source: PLoS One. 2025 Jun 24;20(6):e0326342. doi: 10.1371/journal.pone.0326342 (PMC12186877; doi:10.1371/journal.pone.0326342)
Supplement: Attachment — Submitted filename: reply_reviewers_250319.docx [file pone.0326342.s003.docx]

**Review Comments to the Author**

**Reviewer #1:**

**I have reviewed the manuscript by Keller et al. The manuscript is addressing a relevant aspect of the diagnosis of drug resistant TB using the GeneXpert XDR test. It is reporting the Feasibility and acceptability of the test workflow.

I have raised comments to be addressed below:
Major comment
1) The manuscript is about workflow, how ever the content is mainly about acceptability of the test and less of the workflow. The authors should have looked into the proposed workflow of the GeneXpert XDR and conduct a study looking into the feasibility and acceptability of such steps towards GeneXpert XDR testing. For examples, the feasibility and acceptability of testing the same sample as GeneXpert Ultra Vs collecting another sample, on the same day versus on a different day. looking into both lab, clinic and patient workflow. This could be still looked at through the same study population, Lab, clinician and PWTB. If this is not the case, then, word "Workflow" should be removed from the title and the entire writeup of the manuscript revised to concentrate on the "Feasibility and Acceptability of the test only.**

*Reply: We thank the reviewer for this comment and agree that this paper is about the overall implementation of Xpert MTB/XDR and the impact of adding this new test on the work of health care workers. While we, indeed, did not look specifically and separately at each step in the workflow, we did intend to broadly examine clinic workflow. To address the reviewer’s comment, we have removed the word ‘workflow’ from the title and at places in the manuscript where we felt that the term was not necessary to keep the meaning of the sentence. However, where discussing the overall broader impact of the new workflow, we left the term in.*

**Minor comments
1) abstract: L38: the why "especially laboratories" yet the manuscript is about lab personnel, clinician patients**

*Reply: This line concerns background information, and we believe that introduction of the Xpert MTB/XDR test will especially change the workflow in those laboratories that previously did not conduct Xpert testing, as this means a new test being introduced with an additional follow-on (reflex) test that needs to be done and interpreted, and additional results to be shared with clinicians, whereas previously, another sample might have been collected for submission to a reference laboratory.*

**2) The study happened in only 4-months, was this nested in another already existing study or it was the main and primary study? this seems to be a very short time for a multi-country study.**

*Reply: This study was indeed nested into a larger study, as is briefly explained in the abstract (lines 43-44) and in more detail in lines 108-110. The data collection could be done in just 4 months as data were concurrently collected by different teams in each country (2 interviewers per team).*

**3) There is less literature about the concepts of the study under L108-112**

*Reply: Indeed, this paragraph does not contain any references to literature as it pertains to the study itself, explaining the aims of our study. Therefore, no references were added.*

**4) L132-133: were these numbers considered adequate? Usually IDs consider a sample size of 9-17 participants to reach saturation.**

*Reply: The main population of our study were health care workers. Interviews with patients and policy makers were added to enrich our data. We admit that this may not have become very clear from the manuscript. Therefore, we changed lines 132-136 as follows: “...our aim was to reach thematic saturation in interviews with healthcare workers (clinicians, nurses, laboratory staff). In addition, we interviewed PWTB and policy makers to provide additional insight into the impact of the introduction of Xpert-XDR. The intent here was supplemental and not to reach thematic saturation.”*

**5) L143: Very difficult to find interviewers who can speak all languages the participant feels comfortable! may be the language the can understand.**

*Reply: Interviewers were hired on the basis that they could speak the languages of the interviewees. So indeed, they did feel comfortable in the languages used during the interview.*

**6) L146: The interviewers varied in different strength, BUT the study had only 5 interviewers**

*Reply: As explained in lines 141-142, there were six persons conducting the interviews (five interviewers and one data collector). As explained in the next paragraph, all these were trained both in TB and in qualitative data collection skills. During the training, trainees with less experience in TB but strong qualitative interviewing skills were paired with trainees with more experience in TB but less experience in conducting qualitative interviews.*

**7) Results section: The number of people interviewed are higher than those intended as per L132: Did the REC approve the additional numbers of interviewees than those intended for?**

*Reply: In response to comment #4 of this reviewer, the number of intended interviewees has been removed from the methods section as numbers of interviews planned concerned the maximum number of interviews considered to be needed to reach thematic situation, and are thus not essential. The total number of interviews was lower than this maximum in all countries (25 health care workers, 4 persons with TB and 3 policy makers per country): 20, 20 and 21 health care workers, 3 ,4 and 4 PWTB, and 3, 3 and 3 policy makers were interviewed respectively in South Africa, Nigeria and Ethiopia. Final numbers of interviews conducted are listed in Table 2 in the Results section.*

*In direct response to the comment of going above numbers - there was no need for approval as in qualitative research there is not a strict number of interviews required, but rather the aim of reaching thematic saturation. See also our response to comment #4.*

**8) L188-190: Is this section for methods or these are results?**

*Reply: The sentences “The laboratory staff provided insights into the feasibility and acceptability of the GeneXpert instrument and of conducting the Xpert-XDR assay. The clinicians and nurses provided feedback about the acceptability and feasibility of the new workflow” rather describe the type of information to be collected in the interviews than the precise information that was obtained. We have now changed the sentences slightly and moved them to the methods section (lines 154-156) as proposed by the reviewer.*

**9) L209: This should have a sub section i.e "Factors contributed to Acceptability" and avoid repeating "acceptability or Feasibility going forward.**

*Reply: Subheadings have been adapted to reflect this comment of the reviewer.*

**10) L290: "Lower sensitivity for TB detection". Since this study was not a diagnostic accuracy study, there is possibility of this assumption not being true. There are several factors such as sample collection, bacterial load in the sample and processing as you indicate in table 2 that may affect the outcome of the test. I suggest you change to "discordant results" and not lower sensitivity.**

*Reply:* *While we agree with the reviewer that this is not necessarily true, the text describes results directly associated with the responses from the participants, and not our assumptions. Thus, we feel that leaving the wording as is, is most true to the results.*

**Reviewer #2:**

**The manuscript is well-written.
The authors have stated that the data cannot be publicly shared as it consists of individual opinions provided under the condition of deidentification and restricted access to the research team. However, they could consider sharing a checklist of interview questions along with the aggregated qualitative responses in numerical or percentage form.**

*Reply: While the reviewer provides an interesting suggestion which we will consider if the editor thinks this is needed, we considered this not feasible as the first author has left KNCV and no longer has direct access to the database. Instead, we have added all interview guides to the manuscript as a supplemental file (see reference to this in line 160).*

**The GeneXpert MTB/XDR assay is unable to detect resistance to key drugs such as linezolid, bedaquiline (Group A drugs under the new WHO guidelines), pyrazinamide (PZA), and other repurposed second-line drugs. Furthermore, targeted NGS from clinical specimens is not yet feasible in many low- and middle-income countries (LMICs) with a high burden of DR-TB, making phenotypic DST (pDST) or molecular PCR assays essential for detecting susceptibility or resistance to these drugs. Therefore, it is crucial to assess the turnaround time and cost-effectiveness of using GeneXpert MTB/XDR in combination with pDST or additional molecular PCR assays for drugs like PZA, bedaquiline, and linezolid in XDR-TB patients.**

*Reply: We agree with the reviewer that this is a major limitation of the Xpert-XDR assay, although it was not raised as a limitation by the interviewees. We therefore included this as a limitation in the Discussion section (lines 398-407). To strengthen the manuscript, we now added to the concluding section (lines 443-447): “Finally, it should be noted that additional tests, such as pDST or (targeted) genome sequencing are needed to detect resistance to key drugs as bedaquiline and linezolid. Therefore, it is crucial to assess turnaround times and cost-effectiveness of combining Xpert-XDR which such tests, especially for pre-XDR-TB patients.”*
