# Supplementary material for: Feasibility and acceptability of GeneXpert MTB/XDR implementation among healthcare workers in three low-middle income African countries
Source: PLoS One. 2025 Jun 24;20(6):e0326342. doi: 10.1371/journal.pone.0326342 (PMC12186877; doi:10.1371/journal.pone.0326342)
Supplement: Attachment — Submitted filename: reply_editor_250514.docx [file pone.0326342.s004.docx]

**Response to editor’s comments:**

**1) For Reviewer 1 question 3 about the reference, I believe the reviewer is intending to ask if your proposed workflow is supported by any other published literature? and if so, please give a reference although we understand this is your protocol. however, if this protocol is completely novel to this study, you will have to explain the need for deviation from similar published studies.**

*We apologize the comment of the reviewer. Re-reading the comment (“****There is less literature about the concepts of the study under L108-112****”) as well as the respective paragraph, we understand that the reviewer wants to know if there is any literature underlying the theoretical framework of acceptability and feasibility tha. The theoretical framework of feasibility and acceptability for this study was based on work published by Sekhon et al. (reference 14 in the original manuscript).*

*With regards to the introduction of the Xpert MTB/XDR test in the laboratory workflow, this is based on recommendations by the manufacturer. This is further explained in a publication of the TRiAD study protocol (which was added to the original manuscript as reference 23).*

*We have added references to both papers in lines 108-114 as follows: “The TriAD study aims to evaluate the effectiveness, feasibility, acceptability and cost-effectiveness of implementing a rapid triaging algorithm with the use of Xpert-XDR and selection of all-oral regimens for DR-TB in South Africa, Ethiopia, and Nigeria (13). To assess the programmatic and operational aspects of introducing this algorithm, we assessed its feasibility and acceptability, using the theoretical framework of acceptability of healthcare interventions developed by Sekhon et al. (14). We included barriers and facilitators from the perspective of healthcare workers, policy makers and PWTB in South Africa, Ethiopia, and Nigeria.”*

*All references have been renumbered accordingly.*

**2) As for the reviewer 2's concern about the data sharing, please note that PLOS journals require authors to make all data underlying the findings described in their manuscript fully available without restriction when at all possible. The data should be part of the institution not the author who has left. Please make every effort to make all data available.**

*We do apologize for our confusing answer; as we failed to update it in line with the text we added to the Data Availability Statement. Here, we do explain that the data is available on request and that it can be made available by addressing the respective ethical review boards: “The qualitative data collected for this study contains sensitive information that could lead to the identification of patients, staff and policy makers, despite the data having been de-identified. Data access requests can be made:*

- *For South Africa, Caprisa sites, via the Biomedical Research Ethics Committee of the University of Kwazulu-Natal at the following e-mail address:BREC@ukzn.ac.za, citing protocol reference number BREC/00002654/2021; for the University of Witwatersrand via the Human Research Ethics Committee at the following email address: EthicsRegulatory@witshealth.co.za, citing protocol reference number 210805;*
- *For Nigeria via the National Health Research Ethics Committee of Nigeria at the following email address: secretary@nhrec.net, citing NHREC approval number NHREC/01/01/2007-01/06/2023;*
- *For Ethiopia via the Ethiopian Public Health Institute Institutional Review Board at the following email address: info@ephi.gov.et, citing the EPHI-IRB and protocol reference number EPHI-IRB-384-2021”*
